# FoxP1 marks medium spiny neurons from precursors to maturity and is required for their differentiation

**DOI:** 10.1016/j.expneurol.2016.05.002

**Published:** 2016-08

**Authors:** S.V. Precious, C.M. Kelly, A.E. Reddington, N.N. Vinh, R.C. Stickland, V. Pekarik, C. Scherf, R. Jeyasingham, J. Glasbey, M. Holeiter, L. Jones, M.V. Taylor, A.E. Rosser

**Affiliations:** a Brain Repair Group, Sir Martin Evans Building, School of Biosciences, Cardiff University, Museum Avenue, Cardiff CF10 3AX, United Kingdom; b Molecular Biosciences Research Division, Sir Martin Evans Building, School of Biosciences, Cardiff University, Museum Avenue, Cardiff CF10 3AX, United Kingdom; c MRC Centre for Neuropsychiatric Genetics and Genomics, School of Medicine, Cardiff University, Cardiff CF14 4XN, United Kingdom; d Department of Obstetrics and Gynaecology, School of Medicine, Cardiff University, Cardiff CF14 4XN, United Kingdom; e Central European Institute of Technology (CEITEC), Institute of Anatomy, Masaryk University, A1/064, Kamenice 3, 625 00 Brno, Czech Republic

**Keywords:** DARPP-32, dopamine and cyclic adenosine 3′, 5′- monophosphate-regulated phosphoprotein, 32 kDa, FoxP1, Forkhead box protein P1, HD, Huntington's disease, MSN, medium spiny projection neuron, FoxP1, DARPP-32, Medium spiny neurons, Neural transplantation, Huntington's disease

## Abstract

Identifying the steps involved in striatal development is important both for understanding the striatum in health and disease, and for generating protocols to differentiate striatal neurons for regenerative medicine. The most prominent neuronal subtype in the adult striatum is the medium spiny projection neuron (MSN), which constitutes more than 85% of all striatal neurons and classically expresses DARPP-32. Through a microarray study of genes expressed in the whole ganglionic eminence (WGE: the developing striatum) in the mouse, we identified the gene encoding the transcription factor Forkhead box protein P1 (FoxP1) as the most highly up-regulated gene, thus providing unbiased evidence for the association of FoxP1 with MSN development. We also describe the expression of FoxP1 in the human fetal brain over equivalent gestational stages. FoxP1 expression persisted through into adulthood in the mouse brain, where it co-localised with all striatal DARPP-32 positive projection neurons and a small population of DARPP-32 negative cells. There was no co-localisation of FoxP1 with any interneuron markers. FoxP1 was detectable in primary fetal striatal cells following dissection, culture, and transplantation into the adult lesioned striatum, demonstrating its utility as an MSN marker for transplantation studies. Furthermore, DARPP-32 expression was absent from FoxP1 knock-out mouse WGE differentiated *in vitro*, suggesting that FoxP1 is important for the development of DARPP-32-positive MSNs. In summary, we show that FoxP1 labels MSN precursors prior to the expression of DARPP-32 during normal development, and in addition suggest that FoxP1 labels a sub-population of MSNs that are not co-labelled by DARPP-32. We demonstrate the utility of FoxP1 to label MSNs *in vitro* and following neural transplantation, and show that FoxP1 is required for DARPP-32 positive MSN differentiation *in vitro*.

## Highlights

• FoxP1 was the most highly up-regulated gene during MSN-neurogenesis.
• FoxP1 expression persists in the striatum through development to adulthood.
• FoxP1 is retained in WGE cells in culture and after neural transplantation.
• Striatal DARPP-32 expression is lost in a mouse FoxP1 knock-out.

## 1 Introduction

Medium spiny neurons (MSNs), which comprise approximately 85% of striatal neurons in the mouse, are dysfunctional in a number of neurological conditions. In Huntington's disease (HD) MSNs become impaired and degenerate over a period of several decades (Harper, 1996). This results in progressive motor, cognitive and psychiatric disturbances leading to progressive decline in functioning over a period of 20–30 years, eventually necessitating full-time nursing care. Replacement of damaged striatal cells and reconstruction of striatal circuitry is being actively explored as a therapeutic strategy in this condition (Kelly et al., 2009). Indeed, both animal studies and pilot human transplantation trials have demonstrated functional benefit of this approach (Rosser et al., 2011). A fundamental requirement of transplanted cells is that they accurately differentiate to an MSN phenotype (Precious and Rosser, 2012); dopamine and cyclic adenosine 3′, 5′- monophosphate-regulated phosphoprotein, 32 kDa (DARPP-32) has been widely used as the ‘gold standard’ marker of the terminally differentiated MSN (Walaas and Greengard, 1984, Tamura et al., 2004). However, DARPP-32 is not expressed in MSN precursors and is unreliably detected in culture, perhaps due to insufficient maturity of the emerging post-mitotic MSNs, and is thus a poor marker of developing MSNs.

The only donor cells shown unequivocally to confer functional benefit in HD are those derived directly from primary fetal whole ganglionic eminence (WGE) (Rosser et al., 2011), the WGE being the structure from which the striatum develops. However, fetal tissue availability is limited, and thus there is intense effort to identify a renewable source of donor cells for cell-replacement strategies, with the most likely being one or more sources of stem cells. To date, a small number of protocols have been published for generating MSN precursors from human stem cells (Aubry et al., 2008, Ma et al., 2012, Carri et al., 2013, Arber et al., 2015). Following transplantation into animal models of HD, despite the presence of DARPP-32 positive neurons in the grafts, all of these protocols have been found to be associated with deficits in terms of tissue overgrowth and/or limited functional recovery (Aubry et al., 2008, Ma et al., 2012, Carri et al., 2013, Arber et al., 2015). This raises the question as to whether the expression of currently available markers, including the mature MSN identifier DARPP-32, provides sufficient indication of a population of “genuine” striatal-derived MSNs (Precious and Rosser, 2012). Thus, there is a pressing need to extend our understanding of striatal development and to identify well-validated markers that can indicate the presence of both precursor and mature MSNs. As a step in this direction we carried out a microarray study of developing mouse WGE over the period of peak MSN neurogenesis. Forkhead box protein P1 (FoxP1) emerged as the most up-regulated gene, and we also demonstrated its expression in the human fetal WGE over an equivalent period of development. Here, we show that FoxP1 is a marker of MSNs from an early progenitor stage, before the appearance of DARPP-32, right through to adulthood. We demonstrate that it can be used as a marker in both dissociated and transplanted fetal neural precursors. Furthermore, through the use of cells from FoxP1 knock-out mice, we demonstrate that the differentiation of the mature DARPP-32 positive MSN phenotype *in vitro* is dependent on FoxP1.

## 2 Materials and methods

### 2.1 Regulated procedures

All animal experiments were performed in full compliance with local ethical guidelines and approved animal care according to the UK Animals (Scientific Procedures) Act 1986 and its subsequent amendments. Human fetal tissue was collected in accordance with the Polkinghorne and Department of Health guidelines and with full ethical committee approval as part of the *South Wales Initiative for Transplantation* (SWIFT) program (Kelly et al., 2011).

### 2.2 Collection of mouse WGE

Mouse WGE was dissected from CD1 pregnant mice (through in-house breeding). Mated females were checked daily for a vaginal plug. The day of plug discovery was recorded as embryonic day (E)0. Embryonic rodent tissue was dissected according toDunnett et al. (1992). For microarray and qPCR analysis, tissue was collected at E12, E14 and E16. WGE tissue was dissected and embryos from each litter were pooled with 3 litters of each gestational age collected. The accuracy of dissection was demonstrated by comparing the expression of Gsx2 (predominantly striatal marker) and Pax6 (predominantly cortical mantle marker) in the presumed WGE and adjacent cortical tissues, and demonstrated successful identification of WGE (unpublished data). For neural transplantation of wild type mouse allografts, CD1 embryos were taken at E14, dissected WGEs were pooled and aliquots of cells were transplanted as below.

For collection of mouse FoxP1 knock-out WGE, time mates were set up between two FoxP1 heterozygote knock-out (FoxP1^+/−^) mice (Wang et al., 2004) maintained on a C57BL/6 background. Embryos were taken at E14, and individual WGE were dissected and collected separately from each embryo. WGE from each embryo were processed individually and a sample of tail was retained for genotyping.

### 2.3 Collection of human fetal CNS tissue

Human fetal tissue ranging in age from 8 to 12 weeks post conception (which corresponds to crown rump length of 22–54 mm) was collected according to Kelly et al. (2011). Striatal primordia were dissected in 0.9% saline solution with addition of 0.6% glucose (Hospital pharmacy), transferred to Hibernate E (Invitrogen) for overnight storage at 4 °C and processed the following day.

### 2.4 Gene array process and analysis

RNA extraction was undertaken using TRIzol and RNeasy mini columns (Qiagen) according to the manufacturer's instructions. *In vitro* transcription of each RNA sample to antisense biotinylated RNA was carried out using the BioArray HighYield RNA Transcript Labelling Kit (Enzo Life Science). Labelled cRNA (15 μg) was hybridised to the Mouse430A Affymetrix GeneChip Array for 16 h at 45 °C, GeneChips were washed in an Affymetrix GeneChip Fluidics Station 400 and scanned with an Agilent GeneArray Scanner. The data were analysed in the R/Bioconductor environment. Quality control analysis was performed on the arrays (using the “Simpleaffy” package (http://bioinformatics.picr.man.ac.uk/simpleaffy/)). The array data were normalized using both the MAS5 and RMA algorithms. For each list, ANOVA tests were performed. The Tukey-Kramer *post-hoc* test was used to determine any significantly differentially expressed genes at a false discovery rate (FDR) of 5%. The lists of significantly differentially expressed genes were then analysed for specific patterns of gene enrichment using the GSA package (http://www-stat.stanford.edu/~tibs/GSA/), using gene ontology (GO) probeset annotations that were obtained from the molecular signatures database (http://www.broadinstitute.org/gsea/msigdb/index.jsp). All microarray raw data can be found online at GEO (http://www.ncbi.nlm.nih.gov/geo/query/acc.cgi?token=ovepqugwtlmftuj&acc=GSE55497) (GSE55497).

### 2.5 Quantitative RT PCR (qPCR)

cDNAs were generated from the same RNA samples used above using Superscript II Reverse Transcriptase (Invitrogen) and qPCR was performed using SYBR green (Finnzymes) and an Opticon Monitor 2 system. qPCR conditions were 95 °C for 15 min, followed by 41 cycles of 95 °C for 30 s, 60 °C for 30s and 72 °C for 30 s. Each qPCR was performed in triplicate, and relative quantification against Gapdh was determined according to the ΔΔC(t) method (Livak and Schmittgen, 2001). For analysis of data, the software Opticon Monitor 3 was used (Biorad) and statistical analysis was carried out using SPSS 16.0. Primer sequences were as follows: mouse FoxP1 CAGCCACCCTCTCTATGGAC and AGCGCATGCTCACTGTTG; and human FoxP1 GCAGTTACAGCAGCAGCACCTCC and CAGCCTGGCCACTTGCATACACC.

### 2.6 *In situ* hybridisation (ISH)

Frozen heads of CD1 mice (E12, E14, E16, postnatal day (P)0, P7 and adult) or human fetal brains (8–12 weeks post conception, crown rump length range 22–54 mm) were sectioned at 30 μm using a cryostat, mounted onto Superfrost charged slides and fixed with 4% paraformaldehyde in phosphate buffer. Sections were treated and hybridised overnight with DIG-labelled riboprobes at 58 °C. Following overnight hybridisation, the slides were washed then blocked for 1 h. After blocking, alkaline phosphatase-conjugated anti-DIG antibody (1:1500; Roche Applied Sciences) was applied for 1 h followed by washing and then developing with NBT/BCIP (Roche) in alkaline phosphatase buffer. All slides were visualised using Wild Heerbrugg Makroskop M420 microscope and MagnaFire 2.1C software.

Riboprobes for FoxP1 were generated from cDNA sequences of extracted RNA (forward mouse primer: ATGCTGGAAAACAGCCGAAA, reverse mouse primer: GTGCTCCTCGTGGGACAAG; and forward human primer: GCAGTTACAGCAGCAGCACCTCC, reverse human primer: CAGCCTGGCCACTTGCATACACC), which were inserted into the cloning vector pCRIItopo (Invitrogen). The site of insertion is flanked by RNA polymerase initiation sites; SP6 and T7 thus allowing for sense and antisense strands to be synthesised. All probes were labelled with DIG (Roche). No staining was seen in any of the sections exposed to the sense probe.

### 2.7 *In vitro* differentiation

For mouse tissue, dissected WGE pieces (from E14 embryos) were transferred to 0.1% trypsin (Worthington, New Jersey, USA) and 0.5% DNAse (Sigma, Dorset, UK) in Hank's balanced salt solution (HBSS) for 20 min at 37 °C. Trypsin inhibitor 0.01% (Sigma) and DNAse 0.05% were added and the tissue was incubated for a further 5 min at 37 °C. Tissue was then washed in DMEM/F-12 (Invitrogen), centrifuged at 1000 rpm for 3 min and the remaining pellet was resuspended in 200 μL DMEM/F-12 and triturated 10–15 times with a 200 μL Gilson pipette to generate a single-cell suspension. Cells were counted using a haemocytometer and trypan blue (Sigma) exclusion was used to assess cell viability.

For human tissue, dissected striatal pieces were incubated in equal volumes of TrypLE express (Invitrogen) and Benzonase (1:10,000) (Merck, UK) for 20 min at 37 °C. Tissue was then washed in saline-glucose solution, centrifuged at 1000 rpm for 3 min and the remaining pellet was resuspended in 200 μL DMEM/F-12 and triturated 10–15 times with a 200 μL Gilson pipette to generate a single-cell suspension. Cells were counted and viability assessed (as above).

For *in vitro* differentiation of both mouse and human cells, dissociated cells were plated onto 13 mm glass coverslips coated with poly-l-lysine at a density of 50,000 cells per well in a 30 μL drop of differentiation medium (DMEM/F-12, 1% PS, 2% B-27 (Invitrogen) plus 1% fetal calf serum (FCS) (Invitrogen)) and were allowed to adhere for 2–3 h before the wells were flooded with a further 500 μL differentiation medium. Cultures were maintained at 37 °C in humidified 5% CO_2_ and 95% atmospheric air, and differentiation medium was replaced with fresh medium every 2–3 days. Cultures were allowed to differentiate for 7 or 14 days. Cells were then washed in phosphate-buffered saline (PBS) (Invitrogen) and fixed in 4% paraformaldehyde in PBS for 20 min.

### 2.8 Immunohistochemical analysis of *in vitro* differentiated cells

Fluorescent immunocytochemistry followed standard protocols with primary antibodies for rabbit anti-FoxP1 (1:500; Abcam), mouse anti-FoxP1 (JC12) (1:500; Abcam), mouse anti-β-III-tubulin (1:1000; Sigma), mouse anti-Map2ab (1:500; Sigma), mouse anti-DARPP-32 (1:20,000; a gift from Prof H. Hemmings), mouse anti-GFAP (1:500; Abcam), rabbit anti-GAD65/67 (1:2000; Millipore), rabbit anti-met-enkephalin (1:15,000; Millipore), rat anti-CTIP2 (1:500, Abcam) and rat anti-BrdU (1:200, Oxford Bio). For double labelling, the two primary antibodies that had been raised in different species were added at the same time. Appropriate fluorescent-labelled secondary antibodies (Life technologies) were applied, followed by the nuclear stain Hoechst. Fluorescent staining was visualised using a Leica DRMBE microscope at 560 nm (red), 494 nm (green) and 346 nm (blue). Cell counts were undertaken at 40 × magnification using a counting grid. For unbiased sampling, 5 fields were chosen at random from which to take counts. Pseudocolour fluorescent images were obtained using Openlab 2.1 image analysis software and colour images were processed using Adobe Photoshop.

### 2.9 Immunohistochemical analysis of whole brain sections

Sections were processed for immunohistochemistry using the free-floating, biotin-streptavidin-horseradish peroxidise method with primary antibodies for anti-FoxP1 (as above), mouse anti-DARPP-32 (as above except at 1:10,000), mouse anti-NeuN (1:4000; Chemicon), mouse anti-parvalbumin (1:4000; Sigma), mouse anti-Nkx2.1 (TTF1; 1:500; DAKO) and rat anti-CTIP2 (as above). Stained sections were visualised using a Leica DRMBE microscope and images were obtained using Openlab 2.1 image analysis software. Anatomical areas were identified using the Interactive Atlas Viewer of the Allen Brain Atlas Website: 2015 Allen Institute for Brain Science. Allen Mouse Brain Atlas [Internet]. Available from: http://mouse.brain-map.org and Allen Developing Mouse Brain Atlas [Internet]. Available from: http://developingmouse.brain-map.org.

### 2.10 Quinolinic acid striatal lesions and neural transplantation

Quinolinic acid is a relatively selective MSN toxin, sparing most of the interneuron populations (Schwarcz and Kohler, 1983, Schwarcz et al., 1983, Beal et al., 1986). Animals (CD1 mice and Sprague-Dawley rats) received unilateral injection of 45 nmol quinolinic acid into the right striatum, and transplantation was carried out at 10–14 days post-quinolinic acid lesion as per Kelly et al. (2007). For mouse E14 WGE transplants into the host adult mouse lesioned striatum, 250,000 cells were resuspended in 2 μL and stereotactically injected over 2 min. For human WGE transplants into the host adult rat lesioned striatum, 500,000 cells were resuspended in 2 μL and stereotactically injected over 2 min.

Animals were housed in a natural light-dark cycle with access to food and water *ad libitum*. All surgery was performed under isofluorane anaesthesia and post-surgery animals were recovered in a warmed recovery chamber and received analgesia by Metacam (Boehringer Ingelheim). All animals were perfused transcardially and fixed using 1.5% paraformaldehyde solution before the brains were removed, post-fixed overnight and transferred to 25% sucrose for cryoprotection. Brains were sectioned at 40 μm on a freezing-stage microtome and sections were stored for subsequent histological analysis.

### 2.11 Statistics

All data were analysed using one-way ANOVA with Tukey-Wallis *post-hoc* test and Student's *t*-test with significance set at 0.05.

## 3 Results

### 3.1 FoxP1 expression is highly up-regulated during striatal neurogenesis and is maintained to adulthood where it is expressed in MSNs

To identify genes whose expression is associated with striatal development a microarray analysis of mouse WGE from E12, E14 and E16 embryos was performed, thus covering the period before, during and after peak striatal neurogenesis (van der Kooy and Fishell, 1987, Deacon et al., 1994, Olsson et al., 1995). The full Affymetrix array data are available online at gene expression omnibus (GEO datasets, NCBI).

**Fig. 1:**
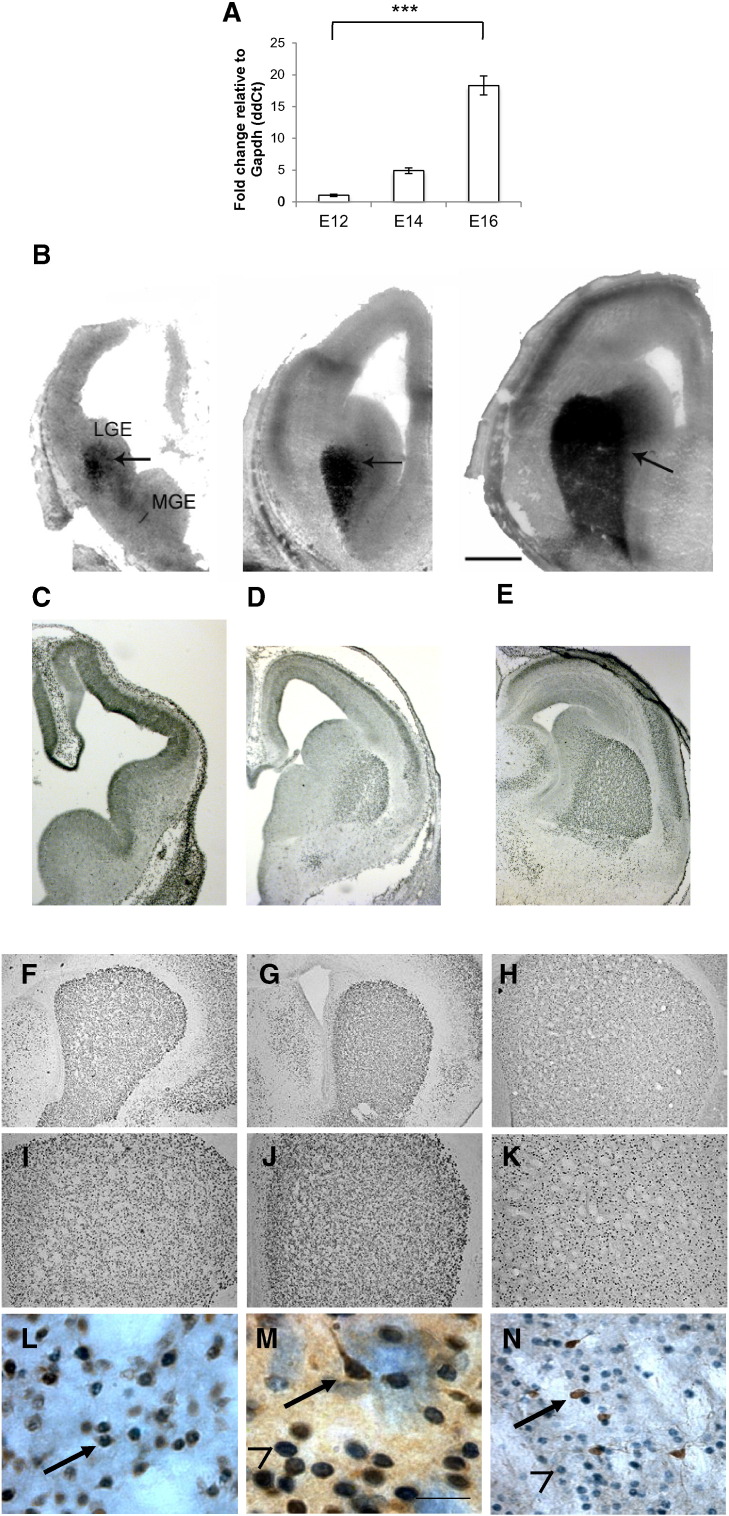
FoxP1 expression in the mouse striatum. Up-regulation of FoxP1 during striatal development in the mouse brain between the ages E12 and E16 was validated using qPCR analysis (A). ISH photomicrographs demonstrate increasing signal intensity and spatial distribution of FoxP1 expression in the region of the WGE from E12 to E16, but not in the subventricular proliferative zone, and there is lower level intensity staining in the cortical plate (B). Photomicrographs of immunohistochemical staining for FoxP1 protein demonstrate a similar expression pattern as shown with ISH in the mouse WGE at E12 (C), E14 (D) and E16 (E). FoxP1 protein expression in mouse brain persists within the striatum through to birth (P0) (F and higher power I), P7 (G and higher power J) and adulthood (H and higher power K). The lower panel of photomicrographs shows adult mouse striatum with immunohistochemical double-staining for FoxP1 (grey)/NeuN (brown), arrow shows double-labelled cell (L); FoxP1 (grey)/DARPP-32 (brown), arrow shows double-labelled cell, arrow head shows FoxP1 positive cell/DARPP-32 negative cell (M); and a lack of double staining with parvalbumin (brown; arrow) and FoxP1 (grey; arrow head) (N). For the graph in (A) *** = *p* < 0.001.

Microarray analysis identified 379 highly up-regulated genes in mouse WGE between E12 and E16, and revealed that FoxP1 was significantly up-regulated between these ages (Table 1). The mouse microarray used in this study had 7 FoxP1 probesets and there was high representation of the FoxP1 probesets in the most up-regulated sequences with FoxP1 appearing 4 times in the top 10, and 7 times in the top 70 probes, based on fold change. Validation of FoxP1 up-regulation was obtained by qPCR analysis of E12, E14, and E16 WGE per unit of tissue relative to GAPDH (Fig. 1A), with an overall significant increase in expression of FoxP1 (F_2,9_ = 98.78, *p* < 0.001).

We then analysed the spatial distribution of FoxP1 at the time points corresponding to those used for the microarray analysis using ISH (Fig. 1B). At E12 expression was restricted to the WGE and absent from the adjacent subventricular proliferative zone. Expression was maintained in the WGE at E14 and E16 as this structure continues to increase in size. By E14 and E16 some staining is also seen in the developing neocortex.

Immunohistochemistry using FoxP1 antibodies in embryonic brain sections demonstrated the presence of FoxP1 protein over the same developmental time period (Fig. 1C-E). Analysis of FoxP1 protein expression was extended into the postnatal period where continued presence of FoxP1 was found in the striatum at P0 and P7 and in the adult striatum (Fig. 1F–K). Fig. 1L–N shows double immunohistochemistry in the adult striatum, which demonstrated that FoxP1 co-localised with both the neuronal marker NeuN (Fig. 1L) and the MSN marker DARPP-32 (Fig. 1M). However, it did not co-localise with a range of interneuron markers (shown for parvalbumin in Fig. 1N). Cell counts within the adult striatum revealed that 100% of FoxP1 positive cells co-labelled with NeuN, although not all striatal NeuN positive cells were FoxP1 positive. 100% of DARPP-32 positive cells co-labelled with FoxP1, but only 86% of FoxP1 positive cells were also DARPP-32 positive. Thus, this reveals a population of FoxP1 positive/DARPP-32 negative cells in the striatum not hitherto reported. To date we have not been able to identify a specific neuronal phenotype marker (including striatal interneuron markers) that identifies this FoxP1 positive/DARPP-32 negative population.

**Fig. 2:**
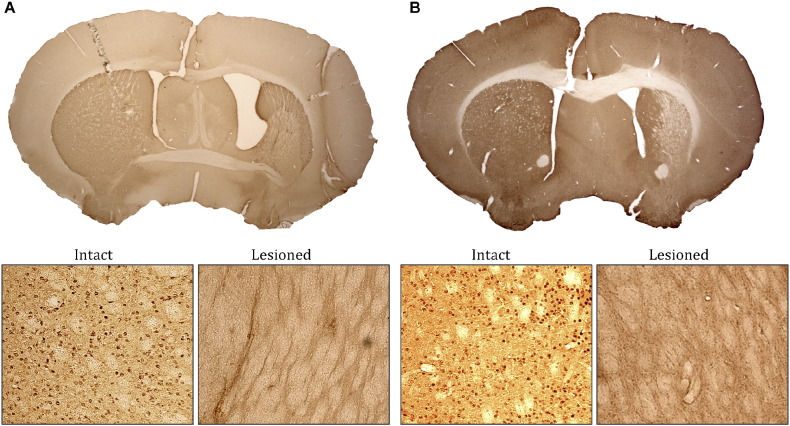
FoxP1 expression in the adult mouse intact and quinolinic acid-lesioned striatum. Immunohistochemistry revealed expression of DARPP-32 (A) and FoxP1 (B) throughout the intact adult striatum (left-hand side of the brain). Following striatal quinolinic acid-lesion, expression of both DARPP-32 (A) and FoxP1 (B) is lost within the lesioned area of the striatum (right-hand side of the brain). The lower panels show higher magnification of the striatal areas.

To confirm the association of FoxP1 with MSNs in the adult striatum, quinolinic acid was used to ablate DARPP-32 positive MSNs (Beal et al., 1986, Davies and Roberts, 1987, Davies and Roberts, 1988). A complete loss of DARPP-32 and FoxP1 in the centre of the quinolinic acid-lesioned area was found (Fig. 2A and B). This demonstrates that the ablation of MSNs, which is associated with a profound loss of DARPP-32 expression, is accompanied by an equivalent loss of FoxP1, thus supporting the notion that FoxP1 selectively labels MSNs in the adult striatum. It is also further confirmation that FoxP1 is not expressed in interneurons, as these are largely spared following the quinolinic acid infusion.

### 3.2 FoxP1 expression is maintained in dissociated primary fetal WGE precursors *in vitro*

**Fig. 3:**
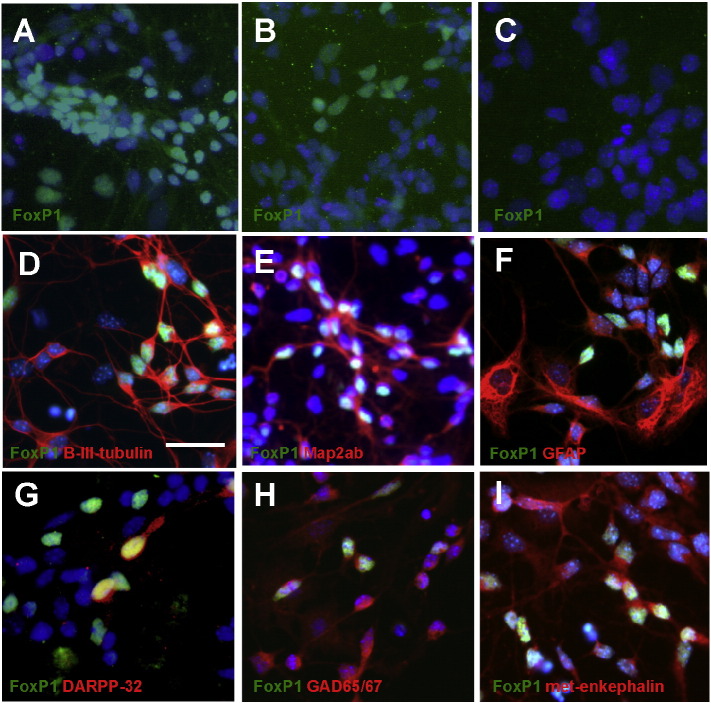
Characterisation of FoxP1 expression in mouse cell culture. Mouse neural tissue from different brain regions was differentiated for 7 days and immunostained for FoxP1 (A–C). FoxP1 expression (green) was seen in cells from the WGE (A) and the cortex (B), but no expression was seen in cells form the ventral mesencephalon (C). Mouse WGE differentiated for 7 days *in vitro*, labelled for FoxP1 (green), which co-expressed the immature neuronal marker β-III-tubulin (red) (D) and the more mature neuronal marker Map2ab (red) (E). There was no co-expression of FoxP1 with the glial marker GFAP (red) (F). FoxP1 positive cells (green) co-expressed the mature striatal MSN marker DARPP-32 (red) (G), the GABAergic neuronal marker GAD-65/67 (red) (H) and the striatal neuronal marker met-enkephalin (red) (I). Nuclei were labelled with Hoechst (blue). Scale bar = 20 μm.

A critical element of neural transplantation protocols is that donor cells are dissociated and suspended in medium in order to be injected into the CNS. Having seen FoxP1 expression in the intact developing brain, we wished to analyse FoxP1 expression in cells that have been dissected, dissociated, and placed in cell culture. To investigate whether the expression of FoxP1 is maintained in differentiating cell cultures, mouse neural cells from wild type embryos at E14 were assessed after seven days differentiation *in vitro* (Fig. 3). FoxP1 positive cells were detected in dissociated cultured WGE (Fig. 3A) and cortex (Fig. 3B), but not in ventral mesencephalon (Fig. 3C) or dorsal mesencephalon (data not shown). The FoxP1 immunopositive WGE cells were identified as neuronal because they co-labelled at early time points in culture with the neuronal marker β-III-tubulin (which is expressed from the start of neuronal maturation), and also with the more mature neuronal marker MAP2ab, but not the astrocytic marker GFAP (Fig. 3D-F). Furthermore, these neurons were identified as the MSN population of cells in these primary fetal mouse cultures through co-localisation of FoxP1 with the striatal MSN marker DARPP-32, the GABAergic neuronal marker GAD-65/67 and the striatal projection neuron marker met-enkephalin (Fig. 3G–I). Notably, as we found in the intact adult brain, although all DARPP-32 immunopositive cells co-expressed FoxP1 there were more FoxP1 immunopositive cells than DARPP-32 immunopositive cells (FoxP1: 40.2 ± 4.0% (mean ± SEM) as a percentage of total cells, and DARPP-32: 8.8 ± 1.0% as a percentage of FoxP1). As mentioned above, the relatively low percentage of DARPP-32 expression in cultured WGE is a common finding that compromises its use as an MSN marker *in vitro*. This finding probably reflects the relative immaturity of the cells *in vitro*. Our interpretation is that FoxP1 labels MSN precursors thus emphasising its utility as a marker of these cells in culture.

### 3.3 FoxP1 is expressed in human fetal WGE

**Fig. 4:**
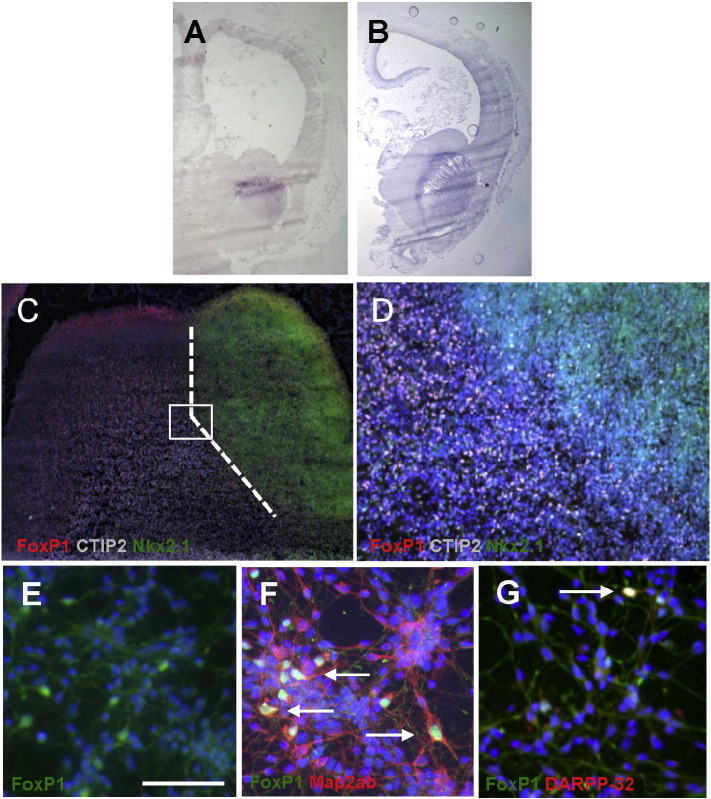
FoxP1 expression in human fetal striatal samples. FoxP1 expression in human fetal brain was assessed using ISH. Two human fetal brain samples were used: crown rump length 26 mm (A) and 52 mm (B). At the earlier time point (crown rump length 26 mm) FoxP1 expression is seen exclusively within the developing striatum (A); by crown rump length 52 mm, FoxP1 expression is visible in the developing cortical region as well as the developing striatum (B). Merged photomicrographs of immunofluorescence carried out on human fetal brain sections from an embryo measuring 48 mm crown rump length (C) and higher power of the boxed area in (C) shown in (D). FoxP1 (red) co-localised with CTIP2 (grey) in the LGE but did not co-localise with Nkx2.1 (green) in the MGE portion of the developing striatum. FoxP1 and CTIP2 double-labelled cells appear white (D). Dashed line in C demarcates the border between the LGE and MGE. Cell nuclei labelled with Hoechst (blue). Human WGE differentiated for 14 days *in vitro* expressed FoxP1 (green) (E); FoxP1 positive cells (green) co-labelled with Map2ab (red) (F); the small number of DARPP-32 positive cells (red) seen co-localised with FoxP1 (green) (G); arrows (in F and G) indicate examples of double-labelled cells. Nuclei were labelled with Hoechst (blue). Scale bar (E–G) = 100 μm.

Having established that FoxP1 is expressed in murine MSNs, both *in vivo* and *in vitro*, we next assessed FoxP1 expression in human WGE at equivalent developmental windows. We analysed spatial expression using ISH on sections from two fetal brains of 26 mm and 52 mm crown rump length (equivalent to approximately 8 weeks and 12 weeks post-conception, respectively, Fig. 4A and B). These show the anatomical distribution of FoxP1 to be in the WGE, and not in the adjacent subventricular proliferative zone. As described above for the mouse, FoxP1 expression was detected in the developing cortical region in the older embryo. qPCR demonstrated FoxP1 expression in 8 separate human fetal striatal tissue samples ranging from 22 to 54 mm crown rump length (equivalent to 8–12 weeks post conception) (data not shown). Immunohistochemical analysis of human fetal brain sections for FoxP1 revealed co-localisation with Coup TF1-interacting protein 2 (CTIP2) (confined to the LGE portion of the developing striatum), but no co-localisation with Nkx2.1 (a classic medial ganglionic eminence marker) (Fig. 4C and D). This provides evidence that the spatial distribution of FoxP1 in the human WGE is similar to the mouse at equivalent developmental stages. Furthermore, FoxP1 was detected in human fetal WGE dissociated cells differentiated for 14 days *in vitro*. Here it co-localised with MAP2ab and DARPP-32; all DARPP-32 positive cells were seen to be FoxP1 positive (Fig. 4E-G), but again, not all FoxP1 positive cells co-expressed DARPP-32. Thus, as in the mouse, we suggest that FoxP1 labels immature MSN precursors that have been dissociated and placed in culture.

### 3.4 FoxP1 expression is maintained in primary fetal WGE precursors transplanted into the quinolinic acid-lesioned striatum

**Fig. 5:**
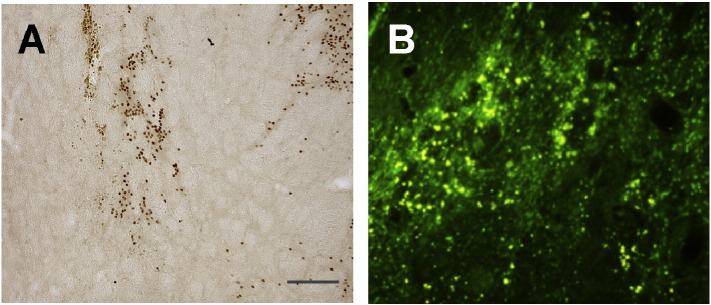
FoxP1 expression post-transplantation. Following transplantation of mouse WGE-derived precursors into the adult mouse quinolinic acid-lesioned striatum, FoxP1 protein (brown) is shown at 4 weeks post-transplant (A). Human fetal WGE-derived precursors transplanted into the adult rat quinolinic acid-lesioned striatum identified FoxP1 positive cells (green) at 20 weeks post transplantation (B). Scale bar (A) = 100 μm.

The ultimate preclinical test for cells differentiated to an MSN phenotype for a therapeutic purpose is to transplant them into an animal model of HD. Therefore, for FoxP1 to be a useful marker in this context, it must continue to be expressed following transplantation of dissociated WGE cells into the adult brain. To assess FoxP1 expression post-transplantation, E14 mouse WGE cells were transplanted into the lesioned side of the mouse host striatum 10–14 days post-unilateral quinolinic acid-lesion. FoxP1 was expressed within the graft area at the first time point assessed, which was 4 weeks post-transplantation and was still present at 12 weeks, with no significant difference in the numbers of FoxP1 positive cells within the grafts at the two survival times (796 ± 144 and 764 ± 276 respectively; t_2_ = 0.10, p = n.s; shown for 4 weeks in Fig 5A). We then asked whether FoxP1 expression was also maintained following transplantation of human WGE cells into the quinolinic acid-lesioned rat striatum. Fig 5B shows FoxP1 cells in the graft area at 20 weeks post-transplantation. Calculation of graft volumes revealed 0.4 ± 0.1 mm^3^ for the mouse-to-mouse transplants, and 13.9 ± 0.9 mm^3^ for the human-to-rat transplants. These results demonstrate that there is continued expression of FoxP1 post-transplantation as the cells differentiate and integrate into the host striatum, supporting the notion that FoxP1 is a reliable marker of developing and mature MSNs for cell replacement studies.

### 3.5 FoxP1 is required for the production of DARPP-32-expressing striatal MSNs

**Fig. 6:**
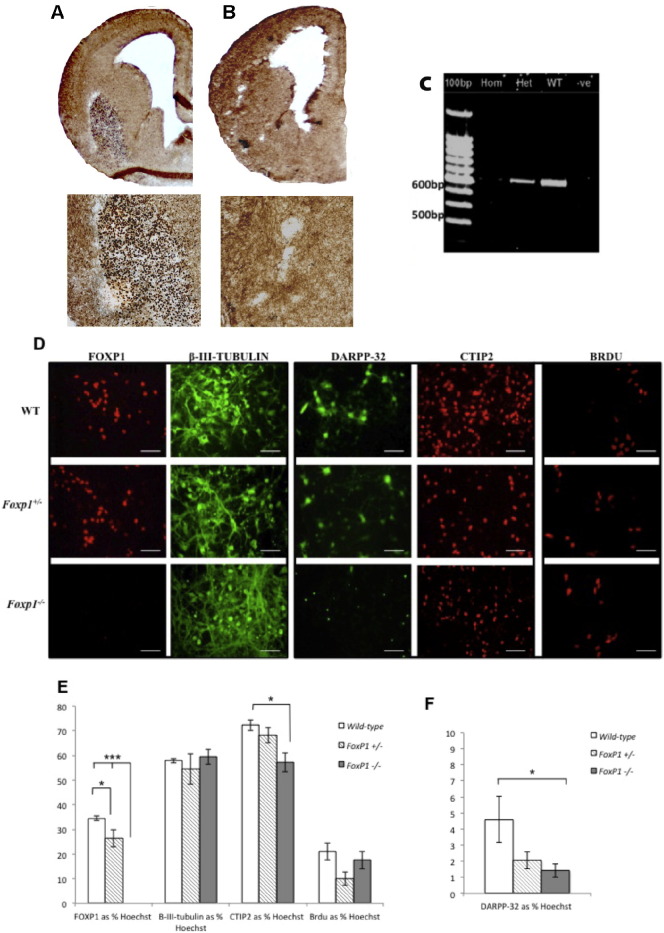
FoxP1 expression in the control and knock-out mouse brain and with *in vitro* analysis after 7 days in culture. FoxP1 immunohistochemical analysis of E14 fetal mouse brain demonstrates FoxP1 expression in the WGE of wild-type (WT) controls (A) and lack of staining in the knock-out mutant (FoxP1^−/−^) (B); lower panels show areas of higher magnification (A–B). RT-PCR of E14 fetal WGE confirms lack of FoxP1 in homozygous embryos (FoxP1^−/−^) and intermediate levels in the heterozygous embryos (FoxP1^+/−^) (C). E14 mouse WGE cultures from each of the three genotypes were differentiated for 7 days. Following fixation cells were double labelled for Foxp1 (red) and β-III-tubulin (green), DARPP-32 (green) and CTIP2 (red). BrdU (red) was also added 24 h prior to fixing to assess proliferation (D). Cells were counted and are represented as a percentage of total Hoechst positive nuclei (E and F). FoxP1 expression was lost in the FoxP1^−/−^ cultures, with no accompanying loss of neuronal numbers. DARPP-32 expression and CTIP2 expression were significantly reduced in the FoxP1^−/−^ cultures, with intermediate levels for the FoxP^+/−^ cultures. There was no significant difference in BrdU expression. Scale bars (D) = 50μm. Each bar on the graphs represents a mean of at least 3 cultures from different embryos and error bars are SEM. Significant *post-hoc* differences are indicated with brackets (ANOVA with Tukey-Wills *post-hoc* test, ***p < 0.001, *p < 0.05).

The previous experiments have demonstrated that FoxP1 is expressed in MSNs from progenitors through to maturity in a range of situations including those relevant for cell transplantation studies. We then wished to ask whether FoxP1 is necessary for the differentiation of DARPP-32 positive MSNs. This question was addressed by using cells from a FoxP1 knockout mouse (Wang et al., 2004). There is loss of FoxP1 expression in the homozygous knockout (FoxP1^−/−^) WGE, and intermediate levels in the heterozygous knockout (FoxP1^+/−^) WGE (Fig. 6A-C). The effect of FoxP1 loss was assessed by comparing the *in vitro* differentiation of E14 WGE cells derived from FoxP1^−/−^, FoxP1^+/−^ and wild type embryos. E14 tissue was used as it is the embryonic stage routinely used for striatal transplantation experiments. E14 is also prior to the *in utero* death of FoxP1^−/−^ embryos, which occurs at E16. Loss of FoxP1 did not appear to affect numbers of neurons (as indicated by β-III-tubulin) or proliferation (according to BrdU expression) (Fig. 6D–E). However, DARPP-32 expression and CTIP2 expression were significantly reduced in the FoxP1^−/−^ cultures (Fig. 6D-F). There was no significant difference between wild type and FoxP1^+/−^ cultures with respect to DARPP-32 and CTIP2 expression (Fig. 6D-F).

In summary, cells lacking FoxP1 are substantially compromised in their ability to differentiate into cells that express the MSN marker DARPP-32, strongly suggesting that FoxP1 is required for the normal differentiation of these neurons.

## 4 **Discussion**

We present evidence that FoxP1 is a valuable marker of both undifferentiated and mature MSNs and, significantly, that FoxP1 is suitable to recognise these cells in transplant paradigms. Furthermore, we have shown that DARPP-32 positive MSNs fail to develop in the absence of FoxP1, indicating that FoxP1 is important for the normal differentiation of striatal MSNs.

Microarray analysis allows an unbiased assessment of gene expression change and using this approach we found that FoxP1 is the most prominently up-regulated gene during peak striatal neurogenesis in the mouse. ISH between E12 and E16 in the mouse revealed intense *FoxP1* staining in the WGE and also expression in the cortex, and similar findings were evident in the human over an equivalent developmental window. We further showed FoxP1 protein expression in the WGE and also demonstrated its continued presence into the neonatal period (P0 and P7) as well as in the adult striatum. The finding of FoxP1 in both fetal and adult brain is consistent with previous studies (Ferland et al., 2003, Tamura et al., 2003, Tamura et al., 2004, Arlotta et al., 2005, Hisaoka et al., 2010, Urban et al., 2010; Allen Mouse Brain Atlas; Allen Developing Mouse Brain Atlas), although demonstration of persistent expression in neonatal brain has not been previously reported.

In our study, the majority of neurons in the adult mouse striatum were FoxP1 positive, and all DARPP-32 positive cells co-labelled with FoxP1, suggesting that FoxP1 labels all MSNs. Our study is consistent with the finding of Arlotta et al, who reported that FoxP1 in the mouse striatum co-labelled entirely with CTIP2, which in turn co-labelled with all DARPP-32 positive cells (Arlotta et al., 2008). Interestingly, we also revealed a population of FoxP1 positive/DARPP-32 negative cells. The absence of any co-labelling of FoxP1 with any interneuron marker is in agreement with Arlotta et al. (2008) and makes it unlikely that these cells are interneurons. Thus, although their identity is not known, we believe that this population is most likely to be either MSNs that do not express DARPP-32 or non-MSN projection neurons.

Further evidence for the association of FoxP1 with striatal MSNs, and not interneurons, comes from the demonstration that FoxP1 expression is completely lost in the quinolinic acid-lesioned mouse striatum. This was the case, not only in the centre of the lesion where, it could be argued, the highest concentrations may result in loss of some interneuron populations, but also in the penumbra of the lesion where we would expect selective loss of MSNs with relative sparing of interneurons (Davies and Roberts, 1987, Davies and Roberts, 1988). This further strengthens the validity of FoxP1 as an MSN marker. Furthermore, the complete loss of DARPP-32 expression with concurrent loss of all FoxP1 supports the notion that the FoxP1 positive/DARPP-32 negative population are either DARPP-32 negative MSNs or non-MSN projection neurons.

Given the strong association of FoxP1 with MSNs from their birth through to adulthood, we suggest that FoxP1 is a valuable marker of both mature MSNs and their progenitors. In the developing mouse brain, DARPP-32 mRNA is undetectable at E14, and even by P0 both DARPP-32 mRNA and protein are present only in very small amounts (unpublished observation and Ehrlich et al., 1990). In contrast to FoxP1, this makes DARPP-32 poorly suited to detecting MSN precursors, and also explains the low percentage of DARPP-32 positive neurons reported in the differentiated cultures here and elsewhere.

There are a number of applications of this increased understanding of FoxP1 in striatal development. The practical use of FoxP1 as an MSN marker in fetal WGE transplantation for HD requires that it continues to be expressed in MSN precursors following dissociation and culture of the donor cells and once such cells are transplanted into the adult striatum. Here we have shown that FoxP1 is expressed in dissociated cultured mouse and human WGE, exclusively in neurons and co-localised with known striatal markers, including DARPP-32. For the reasons discussed above, the proportion of DARPP-32 positive cells in cultured WGE was low, but nevertheless all the DARPP-32 positive cells co-expressed FoxP1. Similarly, fetal WGE from both mouse and human transplanted into the quinolinic acid-lesioned striatum also expressed FoxP1. This provides the basis for the use of FoxP1 as a marker of MSN precursors in such transplantation studies.

For the generation of donor cells from stem cell sources, the poor and unreliable staining of DARPP-32 *in vitro* is also a significant problem. Generation of precursors capable of producing an authentic MSN phenotype is critical to the success of transplantation therapy in HD (Rosser et al., 2011, Evans et al., 2012, Precious and Rosser, 2012). To date most such protocols require multiple steps over many days *in vitro*, and the ability to identify partially specified MSN precursors prior to DARPP-32 expression is a distinct advantage. However, despite the many advantages of using Foxp1 for labelling precursor and mature MSNs, in common with other striatal markers (such as CTIP2 and DARPP-32), it is also found extrastriatally in developing and adult brain (Ferland et al., 2003, Hisaoka et al., 2010; Allen Developing Mouse Brain Atlas; Allen Mouse Brain Atlas; our unpublished findings). Thus, its most powerful use will be in combination with other MSN markers.

Furthermore, a better understanding of normal MSN development will facilitate the optimisation of protocols more likely to produce differentiated cells with a full complement of MSN features. The striking expression of FoxP1 associated with MSN differentiation suggests that FoxP1 might be an important element in the MSN differentiation pathway. We addressed this by analysing the effect of FoxP1 loss on the differentiation of MSNs using WGE cells from FoxP1 knock-out E14 mouse embryos (FoxP1^−/−^). The lack of DARPP-32 staining in these cells cultured *in vitro* strongly implicates FoxP1 as a key element in the differentiation of DARPP-32 positive MSNs. Intriguingly, expression of another transcription factor associated with MSN identity, CTIP2, was reduced but not completely abolished. This raises the question as to the identity of the remaining DARPP-32 negative/CTIP2 positive cells. Full characterisation will require detailed analysis of transplanted FoxP1 knock-out cells to allow maturation beyond what can be achieved *in vitro*, with accompanying analysis of their capacity to undertake any of the functions of the normal MSN population.

In summary, we have presented evidence that FoxP1 is a valuable marker of immature MSNs from an early developmental stage in the mouse, and continues to identify this population throughout life. We also demonstrated the utility of FoxP1 as a marker in both cultured and transplanted MSNs both from mouse and from human. Lastly, the strong association of FoxP1 with differentiating MSNs, and the reduction of DARPP-32 positive cells in cultured FoxP1 knock-out WGE cells, strongly suggests that it has an important role in MSN differentiation.

## Competing interests

The authors declare no competing financial interests.

## Author contribution

AER and MVT conceived the experiments, CMK, SVP, NNV, AEE, RS, VP, RJ, JG, MH, all contributed to the experiments; SVP, CMK, MVT and AER prepared the manuscript. CS was responsible for the procurement of human fetal tissues and LJ assisted in the gene array analysis.

## Figures and Tables

**Table 1:** Gene array probes for Forkhead box protein P1 (FoxP1).

Gene index	E12–E14		E14–E16		E12–E16		FDR corrected *p*-value
Log2 FC	Abs FC	Log2 FC	Abs FC	Log2 FC	Abs FC
1421141_a_at	− 2.0	4.1	− 1.3	2.5	− 3.3	10.1	0.003
1435221_at	− 2.0	4.1	− 1.0	2.1	− 3.1	8.3	0.002
1435222_at	− 1.9	3.6	− 1.2	2.2	− 3.0	8.1	0.004
1455242_at	− 1.9	3.6	− 1.0	2.0	− 2.9	7.3	0.005
1421140_a_at	− 1.6	3.0	− 1.0	2.0	− 2.6	5.9	0.003
1421142_s_at	− 1.4	2.6	− 1.2	2.2	− 2.5	5.7	0.002
1438802_at	− 1.1	2.1	− 1.2	2.3	− 2.3	4.9	0.028
